# Validation of endogenous reference genes in *Buglossoides arvensis* for normalizing RT-qPCR-based gene expression data

**DOI:** 10.1186/s40064-015-0952-4

**Published:** 2015-04-15

**Authors:** Vijay J Gadkar, Martin Filion

**Affiliations:** Department of Biology, Université de Moncton, 18 Antonine-Maillet Ave, Moncton, NB E1A 3E9 Canada

**Keywords:** Ahiflower™, *Buglossoides arvensis*, Corn gromwell, Reference genes, geNorm, NormFinder

## Abstract

Selection of a stably expressed reference gene (RG) is an important step for generating reliable and reproducible quantitative real-time reverse transcription polymerase chain reaction (RT-qPCR) gene expression data. We, in this study, have sought to validate RGs for *Buglossoides arvensis*, a high nutraceutical value plant whose refined seed oil is entering the market under the commercial trade name Ahiflower™. This weed plant has received attention for its natural ability to significantly accumulate the poly-unsaturated fatty acid (PUFA) stearidonic acid (SDA, C18:4n-3) in its seeds, which is uncommon for most plant species. Ten candidate RGs (*β-Act*, *18S rRNA*, *EF-1a*, *α-Tub*, *UBQ*, *α-actin*, *CAC*, *PP2a*, *RUBISCO*, *GAPDH*) were isolated from *B. arvensis* and TaqMan™ compliant primers/probes were designed for RT-qPCR analysis. Abundance of these gene transcripts was analyzed across different tissues and growth regimes. Two of the most widely used algorithms, geNorm and NormFinder, showed variation in expression levels of these RGs. However, combinatorial analysis of the results clearly identified *CAC* and *α-actin* as the most stable and unstable RG candidates, respectively. This study has for the first time identified and validated RGs in the non-model system *B. arvensis*, a weed plant projected to become an important yet sustainable source of dietary omega-3 PUFA.

**Electronic supplementary material:**

The online version of this article (doi:10.1186/s40064-015-0952-4) contains supplementary material, which is available to authorized users.

## Introduction

Gene expression analysis is an important step towards understanding the roles played by genes in the overall cellular and development processes of a plant. It is often used to track changes in gene expression patterns when plants are submitted to different biotic/abiotic conditions. Some of the traditional approaches that have been used in this area include microarray analysis (Schena et al. 1995), differential display (Liang & Pardee 1992), serial analysis of gene expression (SAGE) (Velculescu et al. 1995) and cDNA-amplified fragment length polymorphism (Bachem et al. 1998). These methods are now being increasingly replaced by total transcriptome profiling methods like RNA-Seq analysis (Core et al. 2008; Camarena et al. 2010). Although all these techniques have given useful insights into the mechanistic role played by individual or group of genes in the total transcriptome, they suffer from major drawbacks: they require RNA quantities which, for most approaches listed above, can be difficult to obtain from some biological samples and, in most cases, they involve a laborious workflow.

An alternative approach resulting from the implementation of the classical polymerase chain reaction (PCR) into a fluorescent based format, commonly referred to as the quantitative real-time reverse transcription polymerase chain reaction (RT-qPCR), was developed. This technique has the unique ability to detect and simultaneously quantify the amplified cDNA target in real time (Higuchi et al. 1993). RT-qPCR has considerably changed our ability to analyze the expression of individual genes in scale and scope which was not possible before (Gadkar and Filion 2012a). Specific attributes of this technique include its speed, accuracy, a broad dynamic detection range, requirement of extremely small amounts of input template (1–10 ng) and its relative ease of implementation. As a result, it has found wide-scale applications in every aspects of plant biology, especially for those systems which are either difficult to cultivate or extract nucleic acid from. Irrespective of the technique used, accurate gene expression analysis requires careful consideration of certain factor(s) which, if not properly controlled, could compromise the final gene expression data. Some of these factors are: quality/amount of starting material (RNA), sub-optimal primer design, presence of inhibitor(s) in certain tissues and reverse-transcription efficiencies (Johnson et al. 2005). Any variation amongst these factors has been shown to dramatically alter the final expression pattern (Dheda et al. 2005). A simultaneous control of inter-sample factors is one plausible solution to overcome this problem. However, it can be extremely challenging, both empirically and logistically, to accomplish. To overcome these technical difficulties, various correction strategies have been proposed (Huggett et al. 2005), and of them, normalization of the target gene expression data against a reference gene (RG), is seen as the most reliable and logistically feasible option (Vandesompele et al. 2002).

For any gene to qualify as a RG, not only it does have to be (a.) consistently expressed across various experimental treatments/tissues, but also it has to (b.) display expression levels which fall within the same dynamic range of the test gene(s) (Thellin et al. 1999). Identifying individual genes which are able to satisfy these two criteria has proven challenging, leading to the empirical evaluation of numerous candidates. The genes which take part in the cellular housekeeping (HK) role have proven to be the favorite targets for such investigation as in theory they ought to display an invariant expression pattern across tissues/organs. Examples of HK genes include: tubulins (α-β types), elongation factors (*EF-1a*), glyceraldehyde-3-phosphate dehydrogenase (*GAPDH*), ribulose-1, 5-bisphosphate carboxylase oxygenase (*RUBISCO*), ribosomal (*18S* or *25S*), clathrin adaptor complex (*CAC*), and albumins. Many of these genes have been validated in both model and non-model systems like soybean (Jian et al. 2008), pea (Die et al. 2010), maize (Manoli et al. 2012) and rice (Jain et al. 2006; Ding et al. 2004) amongst others. In all these cases, the suitability of a particular or a set of RGs was determined by following its/their expression in various tissues/organelles under different experimental conditions. To facilitate the selection of the most optimal RG gene from a group of candidate genes, the expression data is statistically analyzed using programs which are based on different algorithms. The top four algorithms developed for this purpose are: geNorm (Vandesompele et al. 2002), NormFinder (Andersen et al. 2004), BestKeeper (Pfaffl et al. 2004) and the comparative Δ*C*_*q*_ method (Silver et al. 2006). Although no consensus exists on the choice of which single platform should be used, a quick literature scan of all the validation studies carried out within the last five years, unequivocally indicates the popularity of geNorm and NormFinder over the other statistical approaches. The geNorm algorithm relies on a pairwise comparison approach and evaluates the variations of relative quantity ratios for each gene pair in a set of expression data. On the other hand, the NormFinder algorithm depends on a statistical and mathematical model that not only estimates the overall expression variation of a candidate gene, but also considers variation between the chosen subgroups.

In many model organisms like *Arabidopsis thaliana* (Schmidt et al. 2005), *Medicago truncatula* (Benedito et al. 2008), rice (*Oryza sativa*) (Jiao et al. 2009; Wang et al. 2010), soybean (*Glycine max*) (Libault et al. 2010) or maize (*Zea mays*) (Sekhon et al. 2011), exhaustive gene expression maps have been developed (Kumar et al. 2011; Jin et al. 2013), which are publicly accessible. This has made it easy for anyone to access the candidate RG sequences not only for these plants but also for genomes to which these model plant share sequence homology. However, for non-model systems, an empirical analysis of potential candidates has to be undertaken prior to commencing any expression analysis. One example of such a non-model system is *Buglossoides arvensis* (L.) I. M. Johnston, a member of the plant family *Boraginaceae*. Commonly referred to as corn gromwell, or more recently as Ahiflower™ (http://www.ahiflower.com), this weed plant has evoked high interest amongst researchers for its nutraceutical value, more specifically for its propensity to naturally accumulate in its seeds a non-traditional plant lipid, stearidonic acid (SDA, C18:4n-3). This omega-3 polyunsaturated fatty acid (PUFA) is a key precursor in the biosynthesis of long chain n-3 PUFAs such as eicosapentaenoic acid (EPA, C20:5n-3) and docosahexaenoic acid (DHA, 22:6 n-3). Consumption of these two n-3 PUFAs by humans is known to have significant health benefits, which include improvement in brain, eye, and cardiovascular health (Lenihan-Geels et al. 2013). As the main sources of EPA (C20:5n-3) and DHA (22:6 n-3) are marine-based and are declining (Pauly et al. 2005), new sources of n-3 PUFAs are now being increasingly sought in order to satisfy the ever increasing demand for omega-3. Consumption of SDA, a natural precursor of EPA and DHA, has shown to impart the same health benefits as consuming EPA and DHA (Surette et al. 2004). The discovery of *B. arvensis*, a natural accumulator of SDA, has opened up exciting possibilities of developing an alternative yet sustainable source of n-3 PUFA’s for human consumption (Surette 2013). Commercial production of this crop plant has recently started under the trade name Ahiflower^™^ by Technology Crops International (Winston-Salem, NC).

The current challenges to the research groups working with *B. arvensis* are (a.) to elucidate the genetic mechanism(s) behind the unusual accumulation of SDA in seeds and (b.) identify factor(s) which could enhance its accumulation. Availability of a suitable RG, validated for this plant system, is the first step towards developing a better understanding of targeted gene expression patterns. Being a non-model system, there is no report in the literature of an evaluation of candidate RGs for this plant species. The present study seeks to address this scientific lacuna by (a.) cloning candidate RGs using an established degenerate primer strategy (b.) analyzing the expression pattern of these genes in various organs/tissues under different experimental conditions, followed by (c.) validation analyses to determine the most stably expressed gene to serve as a suitable RG.

## Materials and methods

### Plant material & growth conditions

Seeds of corn gromwell (*B. arvensis* (L.) I. M. Johnston; plant accession No. TC/07/1R) were obtained from Technology Crops International (Winston-Salem, NC) and germinated by exposing them to a cold stratification regimen (12-h light: 12-h dark photoperiod, 15 ± 1°C, 80% humidity). Plantlets, which emerged 4–6 weeks post-initiation, were transferred into four inch diameter pots, filled with soil and grown for an additional 8–10 weeks with regular fertilization, under optimal growth conditions (12-h light : 12-h dark photoperiod, 20 ± 1°C, 80% humidity) in a growth chamber (Conviron, Winnipeg, Canada). The soil for the experiment was sourced from experimental plots located at the Agriculture and Agri-Food Canada S. H. J. Michaud Research Farm (Bouctouche, NB, Canada) and stored at 4°C till use. This soil was characterized as a gleyed podzolic gray luvisol, a subgroup of the Canadian system of soil classification (Agriculture and Agri-Food Canada 1998), with a pH of 5.2, 62% sand, 25% silt, 13% clay, and 2.6% organic matter.

Different plant growth conditions were used, varying in inoculation and temperature treatments. For inoculation treatments, only plants growing at the optimal growth temperature (20 ± 1) were used. Following transplantation, the plantlets were inoculated with a culture suspension (5 ml/plantlet) of the plant growth promoting rhizobacterium (PGPR) *Pseudomonas fluorescens* LBUM677 or not (control). The concentration of the bacterium was adjusted to 1 × 10^8^ bacteria/ml using 0.8% sterile saline solution. Leaves, stems, flowers and seeds were harvested from both PGPR-inoculated and control plants. Leaves and stems were harvested at 4, 6 and 8 weeks post inoculation. Flower and seeds which appeared any time after the 5^th^ week were harvested at the 6^th^ and 8^th^ week sampling point. For temperature treatments, the plants were grown at two different temperatures apart from the optimal growth temperature: 15 ± 1°C (“low”) and 25 ± 1°C (“high”). The leaves were harvested at 4 and 6 weeks post initiation. For all treatments and sampling points, three biological replicates (destructive sampling) were used. A summary of the different treatments and growing conditions is listed in Table 1.

**Table 1:** **Different growth and treatment conditions of** ***B. arvensis*** **used in this present study**

**No.**	**Experimental treatment**	**Sample Description**
1.	Normal growth	Leaves - Uninoculated (4 weeks)
	(20°C)	Leaves - Uninoculated (6 weeks)
		Leaves - Uninoculated (8 weeks)
2.	Normal growth	Leaves + PGPR (4 weeks)
	(20°C)	Leaves + PGPR (6 weeks)
		Leaves + PGPR (8 weeks)
3.	Normal growth	Stems + PGPR (4 weeks)
	(20°C)	Stems + PGPR (6 weeks)
		Stems + PGPR (8 weeks)
4.	Normal growth	Stems - Uninoculated (4 weeks)
	(20°C)	Stems - Uninoculated (6 weeks)
		Stems - Uninoculated (8 weeks)
5.	Normal growth	Flowers - Uninoculated (6 weeks)
	(20°C)	Flowers - Uninoculated (8 weeks)
6.	Normal growth	Seeds - Uninoculated (6 weeks)
	(20°C)	Seeds - Uninoculated (8 weeks)
7.	Normal growth	Flowers + PGPR (6 weeks)
	(20°C)	Flowers + PGPR (8 weeks)
8.	Normal growth	Seeds + PGPR (6 weeks)
	(20°C)	Seeds + PGPR (8 weeks)
9.	Low temperature growth	Leaves - Uninoculated (4 weeks)
	(15°C)	Leaves - Uninoculated (6 weeks)
10.	High temperature growth	Leaves - Uninoculated (4 weeks)
	(25°C)	Leaves - Uninoculated (6 weeks)

### Total DNA & RNA isolation

Total plant DNA was extracted as follows: the explants were macerated in liquid nitrogen and *ca*. 100 mg was suspended in 300 μL of CTAB buffer (2% CTAB, 1.0 M NaCl, 20 mM EDTA, 100 mM Tris-Cl pH = 8.0) in 2.0 mL centrifuge tubes. The suspensions were vigorously vortexed and incubated at 60°C for 30 min. To this, 300 μL of phenol: chloroform: isoamyl alcohol (24:1:1, v/v) was added and vortexed. The tubes were spun at 16,000 g for 10 min at room temperature. The supernatants were carefully transferred into fresh tubes, mixed with equal volume of chilled absolute isopropanol and incubated at room temperature for 5 min. The tubes were spun at 16,000 g for 10 min at RT and the resulting pellet washed with 70% ethanol. Excess amount of alcohol was drained, the pellets vacuum dried and suspended in 100 μL of TE buffer (10 mM, pH = 8.0). The DNA was quantified using a NanoDrop ND-1000 spectrophotometer (NanoDrop Technologies, Wilmington, DE) and stored at −20°C till further use.

Total RNA was extracted from *B. arvensis* using the Trizol RT extraction system (Invitrogen, Carlsbad, CA) as per manufacturer’s instructions. While sufficient biomass of leaves and stems were available for a conventional (i.e. mortar and pestle based) extraction, a different approach had to be adopted for certain tissue/organ types, specifically seeds and flowers. This was primarily due to the low amounts of recoverable biomass which could be obtained on a per plant basis. To overcome this limitation, the seeds/flowers samples were first snap frozen at −80°C in a 1.5 mL Eppendorf tube and then lyzed using a TissueLyser™ II (Qiagen, Valencia, CA) at the following settings: 2 × 1 min@30 Hz. The macerated tissue was then processed normally using Trizol RT (Invitrogen).

The RNA was checked for concentration and purity using a NanoDrop spectrophotometer (NanoDrop Technologies) and assessed for quality using an Experion™ system (Bio-Rad, Mississauga, Canada). Only the RNA samples which obtained a RNA Quality Indicator (RQI) value above 7 and a 260_nm_/280_nm_ ratio between 1.9 and 2.1 were considered for the present analysis. The RNA samples were treated with the Turbo DNase enzyme I (Ambion, Austin, TX) to remove any residual genomic DNA (gDNA). Complete elimination of residual genomic DNA was confirmed by obtaining no amplification product following 40 cycles of PCR amplification cycles targeting the *α-actin* gene (Table 2). RNA samples were adjusted to 150 ng/μL using DEPC treated water and stored at −80°C till further use.

**Table 2:** **TaqMan-based RT-qPCR primer and probes designed for the ten reference genes isolated from** ***B. arvensis***

**Gene symbol**	**Primer and Probe Sequences**	***B. arvensis*** **GenBank Accession no.**	**Amplification efficiency (%)**	***r*** ^**2**^
**Complete description**	**(5´ → 3´)**			
*β-actin*	**βAct-F :** CCGTCGGGCAACTCATAGTT	KJ883538	94.5	0.998
Beta-Actin	**βAct-R :** GCAGGAGCTTGACACTTCCAA			
	**FAM**-TTCTCAATTGATGAGCTGCT-**MGBNFQ**			
*18S rRNA*	**18S-F:** TCACGACCCGGCCAATT	KJ883536	97.8	0.997
18S ribosomal RNA	**18S-R:** CCGATCGTCTCGTCTCTTCTG			
	**FAM**-AGGCCAGGAGCGTAT-**MGBNFQ**			
*EF-1a*	**EF1a-F:** GCCACACCATCACCAGATACAT	KJ883544	91.5	0.986
Elongation factor 1a	**EF1a-R**: CGTTTTGATGGATTGAGTGATACTG			
	**FAM**-ACTGCAAAATAAATCGAC-**MGBNFQ**			
*α-tub*	**TUB-F:** CCTGAGAAACAAGCCTGTTGAGA	KJ920355	100.2	0.999
Alpha tubulin				
	**TUB-R:** CGCAAGTCCCTCGACATTG			
	**FAM**-TGGTGTAAGTTGGGCGC-**MGBNFQ**			
*UBQ*	**UBQ-F:** CTTCCCGGTGAGGGTCTTG	KJ883543	103.2	0.998
Ubiquitin	**UBQ-R:** ACTTGGTGCTCAGGCTTCGT			
	**FAM**-CGAAGATCTGCATACCAC-**MGBNFQ**			
*CAC*	**CAC-F:** TTGACTGTTTGGTTTGGAAGATAAGA	KJ883539	101.6	0.995
Clathrin adaptor complexes	**CAC-R:** TTCCACCTCAGCACTCAATGTAG			
	**FAM**-AATTTCCTGGACAAACTG -**MGBNFQ**			
*GAPDH*	**GAPDH-F:** AATGGAAGCACAATGAACTTAAGGT	KJ883540	99.7	0.990
Glyceraldehyde-3-phosphate dehydrogenase	**GAPDH-R:** CGTACTGGTTTTTCTCCGAAGAG			
	**FAM**-CATGATGAGAAGACCC-**MGBNFQ**			
*PP2a*	**PP2a-F:** TGCCTTGGCTTCTGTTATTATGG	KJ883542	96.8	0.997
Protein phosphatase 2A regulatory subunit A	**PP2a-R:** GCTGTTCGATTGTAGCATCCTTT			
	**FAM**-AATGGCCCCTGTTCTA-**MGBNFQ**			
*α- actin*	**AACT-F:** CAAGGCTAACAGGGAGAAAATGA	KJ883537	104.6	0.995
Alpha-actin	**AACT-R:** TGAATAGCAACATACATAGCAGGAACA			
	**FAM**-CAAATCATGTTTGAGACATT-**MGBNFQ**			
*RUBISCO*	**RUB-F:** AGAATATTGGTGCTAAGTTGGTCAGA	KJ883541	97.9	0.994
Ribulose-1,5-bisphosphate carboxylase oxygenase	**RUB-R:** CCATCCCCAGCCAGATCAT			
	**FAM**-AGCAGCATCAAAGAC-**MGBNFQ**			

MGBNFQ: minor groove-binding non-fluorescent quencher.

### Cloning of partial sequences of candidate reference genes

Ten candidate reference genes were selected for analysis in this study (Additional file 1: Table S1). As no prior sequence information was available for these candidate genes in *B. arvensis*, a degenerate primer strategy was adopted. No specific criteria for selecting a particular RG was used, however genes that were frequently ranked as the best reference genes for a relatively high number of plant species were selected as potential candidates (Nonis et al. 2012). While most of the RG’s selected are commonly used in different plant systems, an uncommonly used gene, RUBISCO (Gohain et al. 2011), was also tested in the present study. PCR was used to amplify these homologous genes from *B. arvensis* using either cDNA or genomic DNA (gDNA) as templates. Amplicons of correct sizes were gel extracted, cloned into the pKRX-TA plasmid vector (Schutte et al. 1997) and sequenced in both directions (T3/T7 primer combination) using the fluorescent dideoxy chemistry on an ABI 3130xl sequencer (Applied Biosystems, Foster City, CA). Post BLASTn analyses (NCBI) (Altschul et al. 1990), sequences of the RGs were deposited in the GenBank (NCBI) database (see accession no. in Table 2).

### Primer design

PCR primers and TaqMan™ probes for RT-qPCR were designed for the ten genes under study (Table 2) using Primer Express™ software version 3.0 (Applied Biosystems). The PCR primers and TaqMan probes were custom synthesized from Integrated DNA Technologies (Coralville, IA) and Applied Biosystems, respectively. The specificity of each PCR primer set/TaqMan^™^ probe was verified by performing BLASTn searches in the NCBI database and also by visualizing qPCR amplicons obtained from various *B. arvensis* samples using conventional agarose gel electrophoresis (data not shown).

### cDNA synthesis

cDNA was synthesized using the TaqMan Reverse Transcription kit (ABI) and a blend of oligo-dT and random primers. Each RT reaction mix contained 4.2 μl of extracted RNA (150 ng/μl), 2.0 μl of 10× RT Buffer, 4.4 μl (25 mM) of MgCl_2_, 4.0 μl (2.5 mM) of dNTPs, 4.0 μl (50 μM) of random hexamers, 0.5 μl (50 μM) of oligo-dT, 8U of RNase inhibitor and 2.5U of Multiscribe RT enzyme for a final volume of 20 μl. The reaction mixture was incubated at 25°C for 10 min/ 48°C for 30 min/ 95°C for 5 min. The cDNA samples were stored at −20°C prior to use.

### qPCR analysis

qPCR reactions were performed in 96-well plates using an ABI 7500 system (Applied Biosystems). Reactions were performed using the 2X iTaq mixture (Bio-Rad, Hercules, CA) in a total reaction volume of 10 μL. The reaction mixture consisted of: 5 μL of 2X iTaq mixture, 0.4 μL each of forward and reverse primer (5 μM), 0.8 μL of TaqMan^TM^ probe (2.5 μM) and 2 μl of cDNA (1/10 dilution) (cycling conditions: 50°C for 2 min, 95°C for 10 min, and then 40 cycles of 95°C for 15 s and 60°C for 1 min). Fluorescence was detected after each cycle. qPCR technical replicates were used for each sample analyzed and the mean value obtained was used for statistical analysis (see below). For each primer set, standard curves made from serial dilutions of cDNA (spanning five orders of magnitude) were used to estimate PCR reaction efficiency (E) using the formula: E(%) = (10^[−1/*slope*]^ − 1) × 100.

### Data and statistical analysis

Gene expression levels were calculated for all individual RGs based on the number of *C*_*q*_, the number of amplification threshold cycles needed to reach a specific threshold level of detection. To analyze the stability of expression and to identify the most suitable RG, two of the most widely used statistical algorithms, namely geNorm v3.5 (Vandesompele et al. 2002) and NormFinder (Andersen et al. 2004), were used. The geNorm algorithm calculates the expression stability value (M) for each gene and then pairwise variation of a given gene with others. All the tested genes were ranked accordingly to their stability in the tested sample sets, and the number of reference genes necessary for an optimal normalization indicated as such. The NormFinder program identifies the gene(s) with optimal normalization among a set of candidate genes. The lowest stability value (SD) indicates the most stable expression with the gene set examined. Therefore, the software ranks the candidate set genes according to the stability of their expression in a given sample set under a given experimental condition.

A method previously described by (Zhu et al. 2012) was used to give a comprehensive ranking of candidate RGs. We first assigned a series of continuous integers starting from 1 to 10 as weight to each reference gene, according to the RGs ranked by each algorithm from the most stable gene to the least stable gene. We then calculated the geometric mean (GM) of each gene weight across the two methods and then re-ranked these RGs. The gene with the lowest GM is viewed as the most stable RG.

## Results

### Selection of candidate reference genes and primer amplification efficiencies

Candidate genes of expected size as amplified using degenerate primers were found to be homologous (75-92%) to other sequences deposited in the GenBank database. Amplicon products of TaqMan assays on different *B. arvensis* cDNA samples when run on standard agarose gel electrophoresis showed the presence of a single band (data not shown). This indicated that the primers were highly specific. Amplification efficiencies were calculated and ranged from 91.5 to 104.6%, with correlation coefficients of R square higher than 0.98 (Table 2). Thus, all primer/TaqMan^™^ probe combinations were gene specific with high amplification efficiencies.

### Expression profiles of reference genes

**Figure 1:**
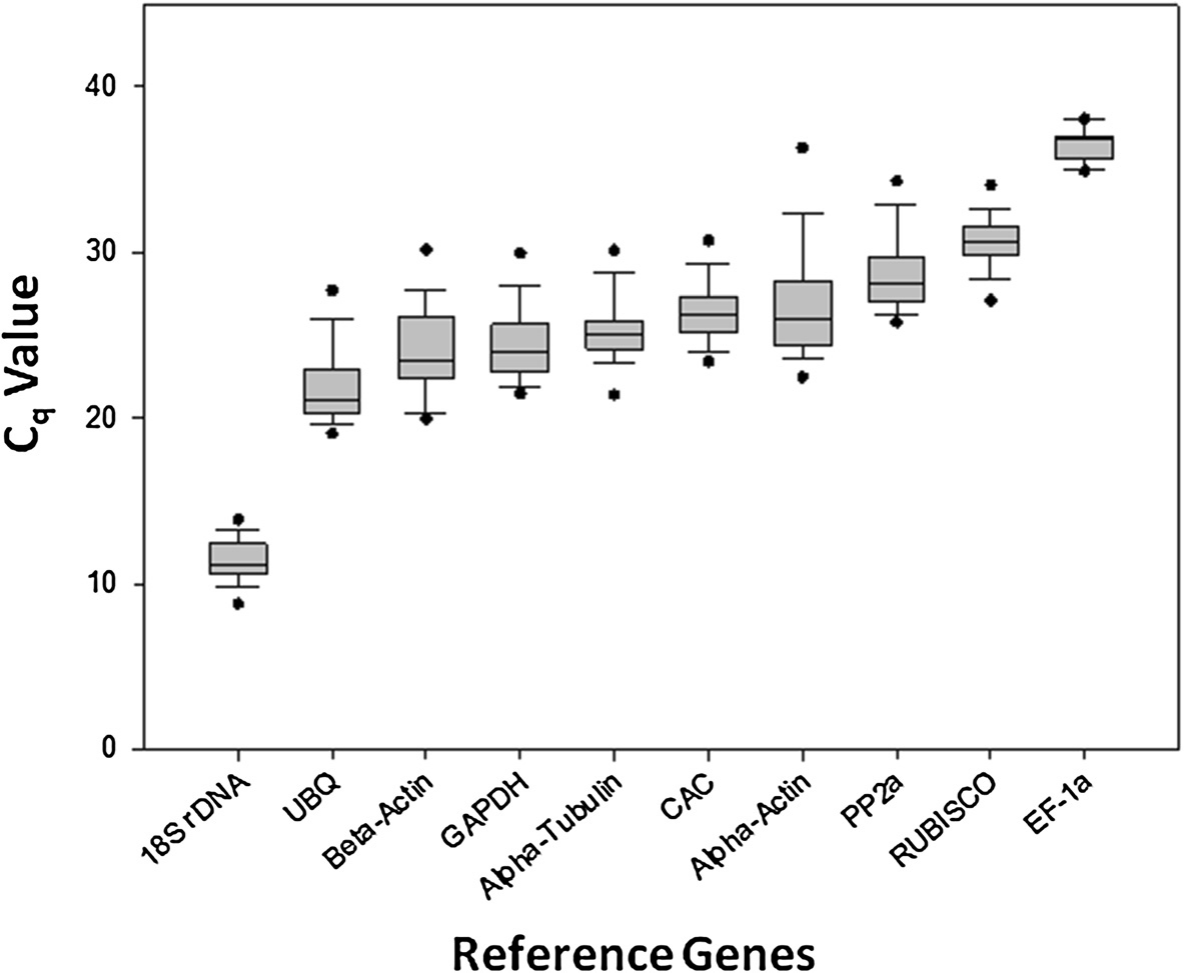
Range of *C*_*q*_ values of the candidate reference genes obtained for all the cDNA samples. Each box corresponds to a specific reference gene as indicated on the x-axis and represents the interquartile range. Whiskers and black dots represent confidence intervals and outliers (5^th^/95^th^ percentile) respectively. The solid line within each box represents the median value.

Some variation in the expression levels of the ten RGs was identified across all samples (pooled treatments and sampling time points) as seen in Figure 1. The *C*_*q*_ values ranged from 9.42 to 35.48 in all the tested samples with the majority of the *C*_*q*_ values being between 19.96 and 29.32 (Figure 1). The gene encoding *18S rRNA* was highly expressed compared to the protein encoding genes, reaching a cycle threshold after only 9.42 amplification cycles, whereas the average *Cq* value of all RGs within the dataset was approximately 27 cycles. As a result, the *18S rRNA* transcript levels were about 2.42 × 10^5^-fold more abundant than the average dataset. The average *C*_*q*_ value of the *EF-1a* gene was 35.48, indicating the least abundant transcripts, followed by the *RUBISCO* gene, with a recorded average *C*_*q*_ value of 30.70. The individual RGs had different expression ranges across all studied sample sets. The lowest range was observed for *EF-1a* while the highest was recorded for the *α-actin* gene respectively. If *EF-1a* was not taken into account, the expression range for all the other genes was between 10.30 and 17.46 cycles.

### Data analysis for expression stability

### geNorm analysis

**Figure 2:**
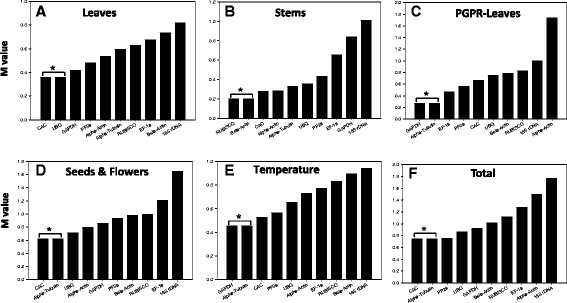
Expression stability and ranking of reference genes as calculated by geNorm analysis in all samples **(F)**, leaves **(A)**, stems **(B)**, PGPR-inoculated leaves **(C)**, seeds & flowers **(D)** and temperature **(E)**. The most stable gene pair identified by geNorm is indicated by an asterix symbol.

The expression stabilities of the 10 RGs were analyzed using geNorm analysis. The tested reference genes were ranked according to stability values (M) and analyzed for specific tissue types, sampling time points and total subsets. According to Hellemans et al. (Hellemans et al. 2007), candidate genes in homogenous sampling panels having M values ≤0.5 are considered stable. However, for heterogeneous samples, M values ≤1 are also acceptable. In the present study, when all samples (heterogeneous sample panel; Figure 2F) were analyzed, the *CAC* and *α-tub* genes (lowest M value of 0.74 each) were found to be most stably expressed while *18S rRNA* (M = 1.28), was the least stably expressed. Although geNorm recommends using 2 RGs, it also calculated the impact of adding additional RGs on normalization (V_n_/_n+1_). Taking into account the entire dataset and considering a cut-off of V_n_/_n+1_ ≤ 0.15 as recommended (Vandesompele et al. 2002), the pairwise value of two genes with the addition of a third one (*PP2a*) (V_2/3_) was 0.13, which could also be used, but for three genes with the addition of a fourth one (V_3/4_), the value obtained was 0.177 and therefore is not recommended for this plant system (Additional file 2: Figure S1).

GeNorm analysis of the individual subset (homogenous sample panel) consisting of leaves (Figure 2A), stem (Figure 2B), PGPR inoculated-leaves (Figure 2C), seeds and flowers (Figure 2D), temperature (Figure 2E), showed some differences compared with the total dataset (Figure 2F). In the first subset composed of leaves (Figure 2A), the *CAC*/*UBQ* (M = 0.35) gene pair was found to be the most stable whereas, *18S rRNA* (M = 0.81) was found to be the most unstable. The low stability of *18S rRNA* was also observed in other data subsets, namely stem (Figure 2B), seeds & flowers (Figure 2D) and temperature (Figure 2E), except in PGPR-inoculated leaves (Figure 2C) where *α-actin* was found to be the most unstable (M = 1.74). Interestingly, *18S rRNA* was the second most unstable gene (M = 0.99). The most stable genes in each of the individual subsets were as follows: *RUBISCO*/*β-act* (M = 0.20) for stem (Figure 2B), *GAPDH*/α-*tub* (M = 0.26) for PGPR-inoculated leaves, CAC/α-*tub* (M = 0.61) for seeds/flowers and finally *GAPDH*/α-*tub* (M = 0.45) for temperature. It should be noted that amongst all the homogenous sampling panels analyzed by the geNorm algorithm, only the seeds and flowers samples gave M (0.61) value >0.5, therefore not within the optimal range as recommended by Hellemans et al. (Hellemans et al. 2007).

### NormFinder analysis

**Figure 3:**
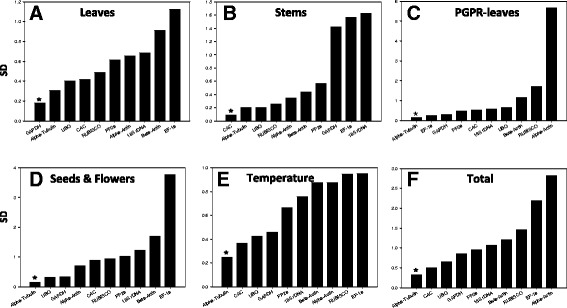
Expression stability and ranking of reference genes as calculated by NormFinder analysis in all samples **(F)**, leaves **(A)**, stems **(B)**, PGPR-inoculated leaves **(C)**, seeds & flowers **(D)** and temperature **(E)**. The most stable gene identified by NormFinder is indicated by an asterix symbol.

The expression stabilities of the 10 RGs were also analyzed using NormFinder analysis. The lowest stability value (SD) indicates the most stable reference gene. The results obtained for NormFinder analysis (Figure 3) were generally similar to those obtained using the geNorm analysis (Figure 2). When all the samples were evaluated, α-*tub* (SD = 0.33), followed by *CAC* (SD = 0.49) were found to be the two most stable genes amongst the ten housekeeping genes analyzed. The α-*tub* gene was also found to be the most stably expressed gene in other subsets, namely PGPR-leaves (Figure 3C), seeds & flowers (Figure 3D) and temperature (Figure 3E). Only in the stem subset was *CAC* found to be the most stably expressed (Figure 3B).

### Comprehensive ranking of candidate reference genes

A side by side ranking of the genes by both the algorithms (Table 3) identified *α-tub* and *CAC* as the most stably expressed genes. The comprehensive ranking results (Table 4) revealed almost the same top seven stably expressed genes, although with slight changes in ranking orders. When considering all samples, *CAC* was identified as the most stably expressed gene and therefore the most suitable RG. *α-actin* was identified as the most unstably expressed gene.

**Table 3:** **Individual ranking of the ten candidate reference genes as identified using geNorm and NormFinder algorithms**

**Experimental sample sets**	**Top two most stably expressed genes**	
	**geNorm**	**NormFinder**
**Leaves**	*CAC*, *UBQ*	*α-tub*, *GAPDH*
**Stems**	*RUBISCO*, *β-actin*	*α-tub*, *CAC*
**Leaves + PGPR**	*α-tub*, *GAPDH*	*α-tub, EF-1a*
**Seeds / Flowers**	*α-tub*, *CAC*	*α-actin, UBQ*
**Temperature**	*α-tub*, *GAPDH*	*CAC*, *α-tub*
**Total Samples**	*α-tub, CAC*	*α-tub, CAC*

**Table 4:** **Comprehensive ranking of the ten candidate reference genes using geNorm and NormFinder algorithms**

**Experimental sample sets**	**Gene expression stability**
	**Most stable** **Least stable**
**Leaves**	*GAPDH, CAC*, *UBQ*, *α-tub, PP2a, RUBISCO, α-actin*, *EF-1a, 18S rRNA*, *β-actin*
**Stems**	*CAC*, *RUBISCO*, *α-tub*, *β-actin, UBQ, α-actin, PP2a*, *EF-1a*, *GAPDH*, *18S rRNA*
**Leaves + PGPR**	*α-tub*, *GAPDH*, *EF-1a*, *PP2a, CAC*, *UBQ*, *18S rRNA*, *β-actin*, *RUBISCO, α-actin*
**Seeds / Flowers**	*α-tub*, *CAC*, *UBQ*, *GAPDH, α-actin, PP2a*, *RUBISCO*, *β-actin*, *18S rRNA, EF-1a*
**Temperature**	*α-tub*, *GAPDH*, *CAC*, *UBQ, PP2a, α-actin, 18S rRNA, β-actin, EF-1a, RUBISCO*
**Total Samples**	*CAC*, *α-tub*, *UBQ, PP2a, GAPDH, β-actin, RUBISCO, 18S rRNA, EF-1a, α-actin*

## Discussion

Over the past decade, several powerful techniques have been developed to detect differences in gene expression levels between different cell types, tissues and organs. Amongst these, RT-qPCR is the most widely used, either to (a.) quantify target gene expressions that are known to get modulated under specific test conditions/biological processes or (b.) as a validation tool for global transcriptome targeting techniques like microarray (Dermatsev et al. 2010) and RNA-seq analysis (Core et al. 2008; Camarena et al. 2010). Under either application scenarios, relative RT-qPCR quantification is the most common methodology used to express the quantum change in the target gene(s) expression profile(s). Inherent to the relative quantification approach is the requirement to normalize the expression of the target gene(s) to the expression of a robust RG, so as to correct for any non-biological variation(s) like sample inconsistencies, operator error and instrumentation/platform differences. Given the extreme sensitivity of RT-qPCR towards such variations, the role played by the RG is now recognized as one of the most important factor towards generating highly reproducible and scientifically interpretable expression data (Dheda et al. 2004).

Choosing a RG is a difficult task since gene expression can not only vary across different tissue/cell types, but also across developmental and physiological states. Ideally, for any gene to qualify as a reference, it should not only be abundant across all tissues/organs, but also be relatively inert to any environmental variations and experimental treatments under investigation. Genes commonly referred to as housekeeping (HK) genes offer the best option to serve as RG as they are expressed in all metabolically active cells/tissues and are critical towards the normal completion of a cell’s life cycle (Warrigton et al. 2000). Predictably, due to their invariant expression profile, HK genes have widely been chosen as valuable normalizing controls in many gene expression analyses including those based on RT-qPCR (Gutierrez et al. 2008; Guénin et al. 2009). Common examples of HK genes are: tubulins, actins, GAPDH, ribosomal subunits and elongation factors amongst others. However, several studies have shown that HK genes are not necessarily expressed at the same level in all tissues (Tricarico et al. 2002; Kouadjo et al. 2007). An unexpectedly high degree of transcriptional variability has also been observed amongst HK genes in different organisms. For example, the *GAPDH* gene, involved in basic cellular functions and often assumed to have a uniform expression pattern, is found to be most stable in barley, oat and grapevine (Reid et al. 2006; Jarošová & Kundu 2010); however its expression in *N. tabacum* has been found to be extremely unstable (Schmidt & Delaney 2010). Another example being that of the *UBQ10* gene, which is stably expressed in the model plant *Arabidopsis* (Czechowski et al. 2005), however not so in other well characterized systems like rice (Jain et al. 2006) and soybean (Jian et al. 2008). Empirical examples like these preclude any arbitrary selection of RGs from the literature or direct transposition of RGs previously identified in one species to another. To avoid such pitfalls, a rigorous empirical examination, validating the stability of candidate RGs, is strongly advised (Dheda et al. 2004) prior to using them in specific experimental conditions. To facilitate this selection process, several statistical algorithms like geNorm (Vandesompele et al. 2002) and NormFinder (Andersen et al. 2004) have been put forward. Any attempts to circumvent the validation process have invariably led to either erroneous results (Tricarico et al. 2002) or gross misinterpretations (Dheda et al. 2005) of the expression data.

In this study, we evaluated ten candidate RGs in *B. arvensis* samples which contained different tissues types, developmental stages and growth conditions. Being a non-model system, this validation process proved challenging as except for few ribosomal subunits encoding genes, no functional genes have ever been sequenced from this plant species. With no prior sequence information available in public databases like GenBank (NCBI) for any functional genes previously identified as potential RGs, the present study focused on addressing this lacuna. Using the well tested strategy of isolating homologous genes using a combination of specific/degenerate PCR primers, several candidate RGs were isolated from *B. arvensis* (Gadkar and Filion 2012b). The choice of these RGs was primarily based on a literature survey where genes belonging to the following classes: tubulins, actins, GAPDH, ribosomal subunits, elongation factors, etc., have successfully been used as RGs in different model (Nicot et al. 2005) and non-model systems (Maroufi et al. 2010; Martin et al. 2008). The identity of the obtained sequences was confirmed using BLASTn analysis (Altschul et al. 1990) and the sequences were then used for designing PCR primers and TaqMan™ probes.

Despite the different statistical approaches used to analyze the expression stability of the ten RGs in *B. arvensis*, we found a general agreement among the methods when considering data from all the samples (heterogeneous sample panel; Figures 2F and 3F). Both of the analytical approaches used, namely geNorm and NormFinder, ranked *CAC* and *α-tub* as the most stably expressed genes, and *18S rRNA* and *α-actin* as the most unstably expressed, for the experimental conditions chosen in the study. Clathrin adaptor complex (*CAC*) subunits link clathrin to their receptors in vesicles, forming a coat, which is important for cargo selection and direction of the vesicle transport (McMahon & Mills 2004). As vesicular transport forms the very basis of endo- and exocytosis, a mechanism through which any plant cell take up nutrients to import signaling receptors or to mediate export of toxic compounds (Alberts et al. 2002), it is not difficult to assume that constitutive expression of *CAC* across different tissues is extremely important for normal functioning and survival. Empirically, this has also been observed during time-course experiments in the model plant *Arabidopsis* (Hong et al. 2010), in *Cucumis sativus* subjected to different nitrogen regimes (Warzybok & Migocka 2013), abiotic stress and exogenously applied growth regulators (Migocka & Papierniak 2011) and in different plant structures of *Fagopyrum esculentum* (Demidenko et al. 2011). Due to its extremely important cellular role, the *CAC* gene has been ranked amongst the top five most stable genes in the model plant *Arabidopsis*. Similarly, the second most stably expressed gene identified in our study, *α-tub*, has been shown to be stably expressed during development in *Orobanche* (Gonzalez-Verdejo et al. 2008), cucumber (Wan et al. 2010), sunflower (Fernandez et al. 2011), and aphid infestation of chrysanthemum (Gu et al. 2011).

The high M (geNorm) and SD (NormFinder) values of the *18S rRNA* and *α-actin* genes respectively, predicting a low stability profile amongst the set of RG’s tested, is consistent with similar observation in many plants. For e.g., the *18S rRNA* gene has been found to be a highly unsuitable normalizer in Chinese cabbage (Qi et al. 2010), pea (Die et al. 2010), *Eucalyptus* spp. (de Almeida et al. 2010) and sunflower (Fernandez et al. 2011), whereas *α-actin* has been found to be an unsuitable RG in *Arabidopsis* (Czechowski et al. 2005), potato (Nicot et al. 2005), rice (Jain et al. 2006), tomato (Exposito-Rodrıguez et al. 2008) and wheat (Paolacci et al. 2009). Interestingly, *18S rRNA* has been used as a reference gene in certain plants (Jain et al. 2006; Nicot et al. 2005), however its use as a normalizer has attracted much controversy (Stürzenbaum & Kille 2001; Thorrez et al. 2008). The primary reason for the unsuitability of the *18S rRNA* gene or any other ribosomal subunit genes in general as a reference gene, is their inherently high expression levels in cells versus functional mRNAs. This hyper expression is believed to result in a stoichiometric imbalance between the rRNA and mRNA fractions, resulting in skewed normalized data. As commonly observed in other studies, we also observed significantly higher levels of *18S rRNA* gene expression (Avg *C*_*q*_ = 11.58) in our experimental system as compared to the other nine functional genes under study (Avg *C*_*q*_ = 27.31). Similarly, *α-actin* which is involved in the basic cytoskeletal functioning was found to be the most unsuitable and moderately unsuitable RG by NormFinder and geNorm, respectively. The fact that members of the actin gene family are influenced by various external factors (Dheda et al. 2005; Thellin et al. 1999; Tricarico et al. 2002) could be a reason for its poor ranking in the stability index.

When the expression stability was analysed separately for each sample set, the ranking for each gene was not always uniform. Some of the variation in expression levels may be due to the role of a specific reference gene in specific tissues. For example, the role of *RUBISCO* in the chloroplast as a component of photosystem I, could explain the low expression levels of this gene in some tissues like seeds and flowers. On the contrary, *UBQ* which is universally expressed was found to be stably expressed across all the tissues, however not up to the levels of *CAC* and *α-tub*. Surprisingly, the *GAPDH* gene involved in the basic cellular functions and often assumed to have a uniform expression pattern in a variety of crop plants like flax (Huis et al. 2010), oat and grapevine (Reid et al. 2006; Jarošová & Kundu 2010) was found not to be very stably expressed in *B. arvensis*. Similar contrasting results have also been observed for this gene in the widely used model plant *N. tabacum* (Schmidt & Delaney 2010). Another gene widely used for normalization, namely *EF-1a*, was also found not to be stably expressed across various tissues and experimental conditions in *B. arvensis*. Similar results for this gene have also been observed in the model test plant *Arabidopsis* (Czechowski et al. 2005), barley, oat, wheat (Demidenko et al. 2011) and in *Salvia miltiorrhiza* (Yang et al. 2010). These results unequivocally confirm that a universal reference gene does not exist, highlighting the need to evaluate commonly used RGs for a particular plant species or a specific experimental condition.

## Conclusions

A significant amount of transcriptional data, including *in silico* analysis, is currently available for major model plant species for validations studies. This considerably enhances the researcher’s ability to identify potential genes for normalization purposes. However, for most non-model plant species, the suitability of any gene as a reference has to be empirically verified, which invariably includes the labor intensive process of mining individual genes. In the present work, we isolated ten potential RGs from the weed plant *B. arvensis*, and empirically validated their stability profiles under different experimental conditions. Our results demonstrated the highly stable expression of *CAC* and *α-tub* genes in different tissues/organs and under different experimental conditions. Certain classically-used reference genes such as *actin* (α and β forms) and *GAPDH* were found to be not necessarily the most suitable RG candidates in *B. arvensis*. In conclusion, we recommend using *CAC* and *α-tub* as suitable RGs in *B. arvensis*, a plant which in the future could become an important source of renewable n-3 PUFA. We however would like to highlight that any researcher interested in studying gene expression in *B. arvensis* under conditions not described in this study should perform validation of candidate gene(s) to ensure optimal normalization. To the best of our knowledge, this is the first report of a validation study in this plant species.

## Additional files

Additional file 1: Table S1. Description of the degenerate primer pairs designed for PCR-based isolation of reference genes from *B. arvensis* (Gonzalez-Verdejo et al. 2008; Lin et al. 2000; Obrero et al. 2011; Ma et al. 2012; Matsui et al. 2004). Additional file 2: Figure S1. Pairwise variation (V) analysis of total samples to determine the optimal number of reference genes required for effective normalization.
