# Supplementary material for: Validation of endogenous reference genes in Buglossoides arvensis for normalizing RT-qPCR-based gene expression data
Source: Springerplus. 2015 Apr 15;4:178. doi: 10.1186/s40064-015-0952-4 (PMC4404469; doi:10.1186/s40064-015-0952-4)
Supplement: Additional file 1: Table S1. — Description of the degenerate primer pairs designed for PCR-based isolation of reference genes from B. arvensis (Gonzalez-Verdejo et al. 2008; Lin et al. 2000; Obrero et al. 2011; Ma et al. 2012; Matsui et al. 2004). [file 40064_2015_952_MOESM1_ESM.docx]

**Table S1** Description of the degenerate primer pairs designed for PCR-based isolation of reference genes from *B. arvensis*

| **Gene symbol** | **Gene name** | **Primer sequence (5´→3´)** | **Reference** |
| --- | --- | --- | --- |
| *α-actin* | Alpha actin | **F**:GTNARYAACTGGGATGAYATGG  **R**: ACAATACCWGTWGTRCGACC | Gonzalez-Verdejo et al. 2008 |
| *18S rRNA* | 18S ribosomal RNA | **F**: AACGGCTACCACATCCAAGG  **R**: CCGAAGGCCAACACAATAGG | Lin et al. 2000 |
| *EF-1a* | Elongation factor-1a | **F**: AAGCTTGATGASTACCTTCTG  **R**: CSACAGAGACNARGTCATCC | http://www.ncbi.nlm.nih.gov/geo/query/acc.cgi?view=data&acc=GPL10120&id=13694&db=GeoDb_blob43 |
| *α-tub* | Alpha tubulin | **F**: TTCAATGCTGTTGGTGGNGG  **R**: TTGGCRTACATSAGRTCGAA | http://www.ncbi.nlm.nih.gov/geo/query/acc.cgi?view=data&acc=GPL10120&id=13694&db=GeoDb_blob43 |
| *UBQ* | Ubiquitin | **F**: GAYTACAACATYCAGARGGAG  **R**: GCRAARATCARCCTCTGCTG | Gonzalez-Verdejo et al. 2008 |
| *CAC* | Clathrin adaptor complexes | **F**: TTYGGGCWAARATGTTTGCGCTKGG  **R**: GGMACCTGRAAYYCCATYTGRATHGG | Obrero et al. 2011 |
| *GAPDH* | Glyceraldehyde-3-phosphate dehydrogenase | **F**: CATGGSSAAGATYAAGATCGG  **R**: AGATGCTKGACCTGYTGTCACC | Ma et al. 2012 |
| *PP2a* | Protein phosphatase 2A regulatory subunit A | **F**: TAAGTCHTGGCGYGTKCGYTAYATG  **R**: ACCTGRTTBACTTGRTCAAGYTTGCT | Obrero et al. 2011 |
| *β-actin* | Beta actin | **F**: GARAARATGACNCARATHATG  **R**: TCNACRTCRCAYTTCATDAT | Matsui et al. 2004 |
| *RUBISCO* | Ribulose-1,5-bisphosphate carboxylase oxygenase | **F**: GGMCCAAARGGAAGGAATGT  **R**: GTTTGTGCTCCMACCTGWAT | http://www.ncbi.nlm.nih.gov/geo/query/acc.cgi?view=data&acc=GPL10120&id=13694&db=GeoDb_blob43 |
