# Supplementary figures and images for: Validation of endogenous reference genes in Buglossoides arvensis for normalizing RT-qPCR-based gene expression data
Source: Springerplus. 2015 Apr 15;4:178. doi: 10.1186/s40064-015-0952-4 (PMC4404469; doi:10.1186/s40064-015-0952-4)

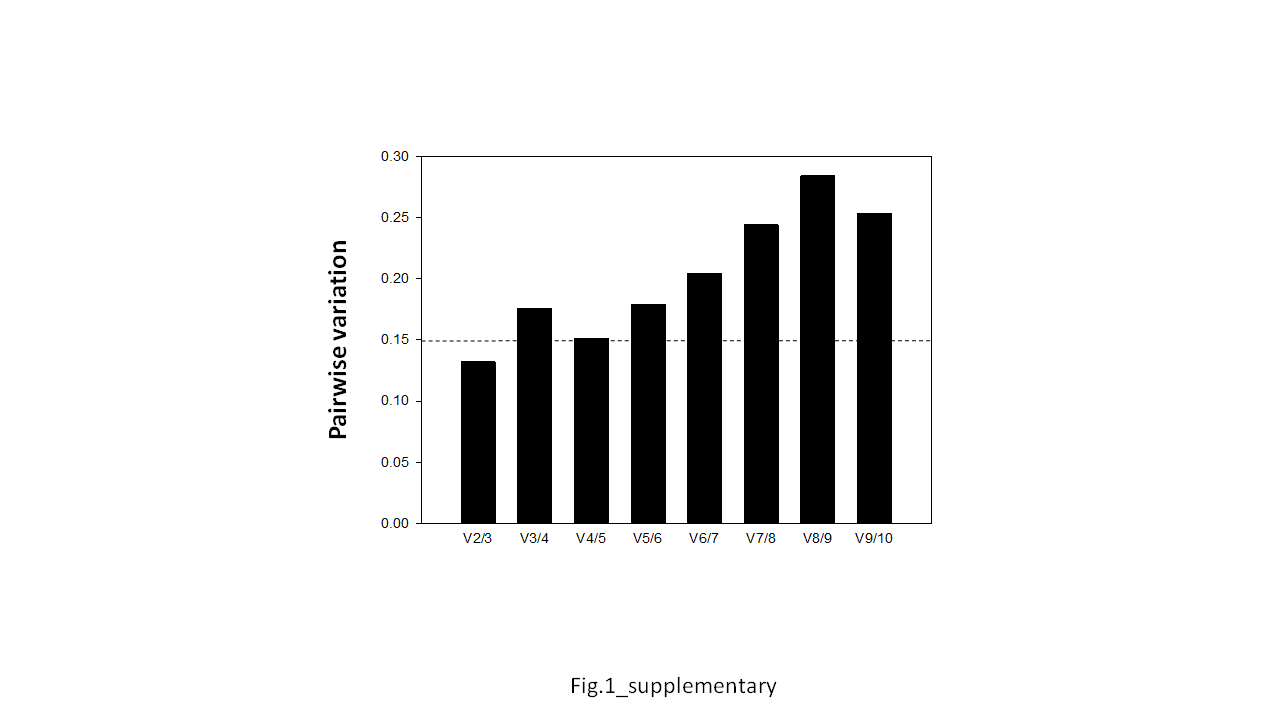

Supplement: Additional file 2: Figure S1. — Pairwise variation (V) analysis of total samples to determine the optimal number of reference genes required for effective normalization. [file 40064_2015_952_MOESM2_ESM.tiff]
